# Terahertz spectroscopy of collective charge density wave dynamics at the atomic scale

**DOI:** 10.1038/s41567-024-02552-7

**Published:** 2024-07-15

**Authors:** Shaoxiang Sheng, Mohamad Abdo, Steffen Rolf-Pissarczyk, Kurt Lichtenberg, Susanne Baumann, Jacob A. J. Burgess, Luigi Malavolti, Sebastian Loth

**Affiliations:** 1 Institute for Functional Matter and Quantum Technologies, University of Stuttgart, Stuttgart, Germany; 2 Max Planck Institute for Solid State Research, Stuttgart, Germany; 3 Max Planck Institute for the Structure and Dynamics of Matter, Hamburg, Germany; 4 Department of Physics and Astronomy, University of Manitoba, Winnipeg, Manitoba Canada; 5 The Manitoba Quantum Institute, Winnipeg, Manitoba Canada

**Keywords:** Electronic properties and materials, Electronic properties and materials, Terahertz optics

## Abstract

Charge density waves are wave-like modulations of a material’s electron density that display collective amplitude and phase dynamics. The interaction with atomic impurities induces strong spatial heterogeneity of the charge-ordered phase. Direct real-space observation of phase excitation dynamics of such defect-induced charge modulation is absent. Here, by utilizing terahertz pump–probe spectroscopy in a scanning tunnelling microscope, we measure the ultrafast collective dynamics of the charge density wave in the transition metal dichalcogenide 2H-NbSe_2_ with atomic spatial resolution. The tip-enhanced electric field of the terahertz pulses excites oscillations of the charge density wave that vary in magnitude and frequency on the scale of individual atomic impurities. Overlapping phase excitations originating from the randomly distributed atomic defects in the surface create this spatially structured response of the charge density wave. This ability to observe collective charge order dynamics with local probes makes it possible to study the dynamics of correlated materials at the intrinsic length scale of their underlying interactions.

## Abstract (web summary)

The observation of phase modes of charge density wave has been a long-standing challenge. Such low-energy phase excitations have now been seen in a transition metal dichalcogenide.

## Main

The uniform charge distribution of many (semi-)metallic materials becomes unstable at low temperatures, and charge ordering emerges^1–3^. Especially at reduced dimensionality, for example, in quasi-one-dimensional (1D) or two-dimensional (2D) metals, the electronic susceptibility is particularly sensitive to periodic perturbations of the atomic lattice so that electron–phonon coupling can induce charge ordering and even cause a metal-to-insulator transition^4,5^.

A distinctive case is the incommensurate charge density wave (CDW), in which phonon softening stabilizes a wave-like modulation of the charge density and the atomic lattice that does not match to the periodicity of the host material^6,7^. CDWs feature new collective dynamics, associated with excitations of the amplitude or phase of its order parameter, that are absent in the normal metal phase. Whereas materials in which commensurability with the host lattice shows phase modes at finite energy^8^, the incommensurate CDW^9^ should feature a Nambu–Goldstone mode that could carry electric current without energy loss by collective phase motion^10^. However, disorder and impurities lead to spatial heterogeneity of the charge-ordered phase^11^ that can strongly modify the collective dynamics^12,13^ and collapse the collective phase mode into phason excitations at very low energies^8^.

Pinning of the CDW by defects leads to insulating behaviour for select 1D incommensurate CDW materials, such as NbSe_3_ (ref. ^14^) or indium atom chains on Si (ref. ^15^), and kinks in the temperature-dependent conductivity of 2D materials in which only part of the Fermi surface becomes gapped in the CDW phase^4,16^. Whereas CDW pinning at individual defects was imaged locally by scanning tunnelling microscopy (STM)^5,17,18^, the defect-induced low-energy phase dynamics have thus far been observed with techniques that averaged over ensembles of defects: neutron scattering found dispersing phase excitations down to 0.25 THz (ref. ^19^); ultrafast low-energy electron diffraction observed slow phase ordering during relaxation from an incommensurate to a nearly commensurate CDW^20^; MHz-range noise in transport measurements indicates phase sliding across pinning potentials^14^; and X-ray diffraction and optical spectroscopy found picosecond-scale relaxation attributed to phase fluctuations^21,22^.

**Fig. 1:**
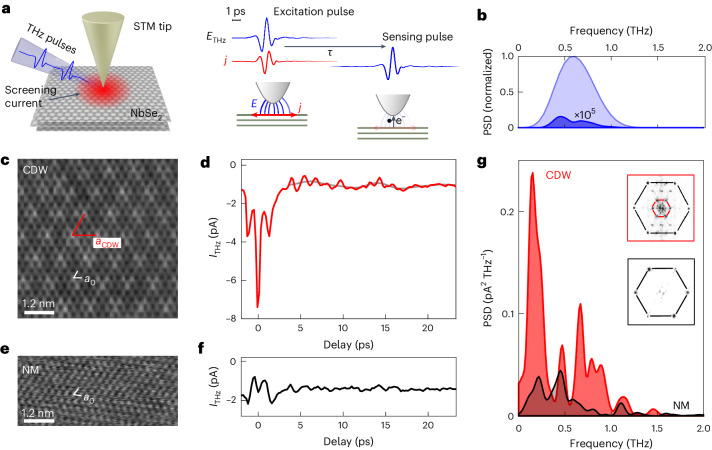
Ultrafast CDW dynamics of 2H-NbSe_2_ on the atomic level. **a**, Left: sketch of the excitation and detection of CDW dynamics in an STM with THz pulses. Right: THz pump–probe detection scheme. One THz pulse excites the sample (excitation pulse) via the screening current (*j*) induced on the sample surface by the THz electric field (*E*_THz_). A time-delayed second pulse (sensing pulse) measures the local dynamics by inducing tunnelling of electrons (e^−^). **b**, PSD of the THz pulse’s electric field between −3 ps and 3 ps (light blue) and of the residual electric field oscillation from 3 ps to 20 ps (dark blue) measured by electro-optic sampling (see Extended Data Fig. 1 for time trace). PSDs are normalized to the THz pulse’s spectral density at 0.55 THz. **c**,**e**, Constant-current topographies of 2H-NbSe_2_ in the CDW phase at 20 K (**c**, junction set point 40 pA at 32 mV) and in the normal metal phase (NM) at 150 K (**e**, junction set point 25 pA at 40 mV) showing the atomic lattice with period *a*_0_ at both temperatures and the CDW with period *a*_CDW_ only at 20 K. **d**, Time trace of the THz-induced tunnelling current recorded in the CDW phase at 20 K (red curve). Junction set point 1 nA at 1 mV. The grey curve shows the low-frequency component of the signal between 0 THz and 0.25 THz obtained by bandpass filtering of the signal’s Fourier transform. **f**, Time trace of the THz-induced tunnelling current recorded in the NM phase at 150 K. Junction set point 2 nA at 1 mV. **g**, PSD of the time traces in the CDW phase (red area) and normal metal phase (NM, black area) in **d** and **f**. Insets: absolute value of the Fourier transform of the STM topographies in **c** and **e**, showing the atomic lattice and CDW peaks in the CDW phase (red box) and only the atomic lattice peaks in the NM phase (black box). Source data

In this Article, we report direct real-space observation of low-energy CDW dynamics in 2H-NbSe_2_ at frequencies ranging from 0.15 THz to 0.9 THz. We use STM to spatially resolve the CDW and excite the tunnel junction with single-cycle THz pulses to measure the CDW’s ultrafast dynamics (Fig. 1a). We find that the oscillating electric field of the THz pulses drives a strong screening current in the NbSe_2_ surface that excites the phase of the CDW locally and creates a spatially heterogeneous pattern of modes by the interplay between THz excitation and pinning at atomic defects.

NbSe_2_ hosts a quasi-2D incommensurate CDW below a critical temperature of 33 K (ref. ^23^) that appears in STM images as a supermodulation of the charge density with average periodicity of 3.06 unit cells of the host lattice^24^ (Fig. 1c). Thus, the CDW’s complex order parameter (given by the amplitude of the charge modulation and its phase relative to the lattice) can be resolved in real space. Static STM shows that atomic impurities are strong pinning sites that locally lock the CDW^17,18^, making NbSe_2_ a good candidate for observing phase excitations that emerge from the interaction of the CDW state with the disorder potential of the randomly distributed defects.

This highly localized dynamics can be detected by exciting the STM’s tunnel junction with pairs of THz pulses: the first THz pulse excites the CDW, and the time-delayed second THz pulse probes the resulting dynamics locally by THz-induced electron tunnelling at a defined time after excitation (Fig. 1a). Sweeping the time delay between the pulses while recording the tunnel current constructs a time trace of the junction conductivity that depends on the ultrafast modulation of the surface charge density. The THz pulses used in this experiment consist of only one optical cycle, which provides a time resolution of better than 0.4 ps in our experiment (Supplementary Section 1). The pulses have a peak electric field strength of 130 V cm^−1^ in the optical far field, which is too weak to alter the static properties of NbSe_2_, but strong field enhancement under the STM tip enables local excitation.

Time traces recorded on NbSe_2_ in the CDW phase at 20 K show an ultrafast relaxation with a time constant as fast as 0.6 ps followed by oscillatory dynamics that persist for at least 20 ps (Fig. 1d). The additional sharp dip–peak structure at zero delay is caused by the overlap of the two THz pulses (see Supplementary Section 1 for details). By contrast, when NbSe_2_ is in the normal metal phase at 150 K, no CDW modulation is detectable in STM images (Fig. 1e) and the time traces exhibit a much weaker response (Fig. 1f). Whereas a dip–peak structure and an ultrafast decay with similar time constant remain present, the PSDs of the oscillatory behaviour at delay times larger than 3.6 ps differ substantially between the normal metal phase and the CDW phase (Fig. 1g).

In the normal metal phase, the spectral weight is concentrated between 0.2 THz and 0.7 THz with two peaks at 0.22 THz and 0.45 THz and a minor peak at 1.1 THz. In the CDW phase, the spectral weight distribution shows prominent additional peaks at 0.15 THz and between 0.6 THz and 0.8 THz. Their spectral weight is more than five times higher than that in the normal metal phase. This indicates the presence of low-energy excitations in the CDW state that are absent when the NbSe_2_ is in the normal metal state.

**Extended Data Fig. 1:**
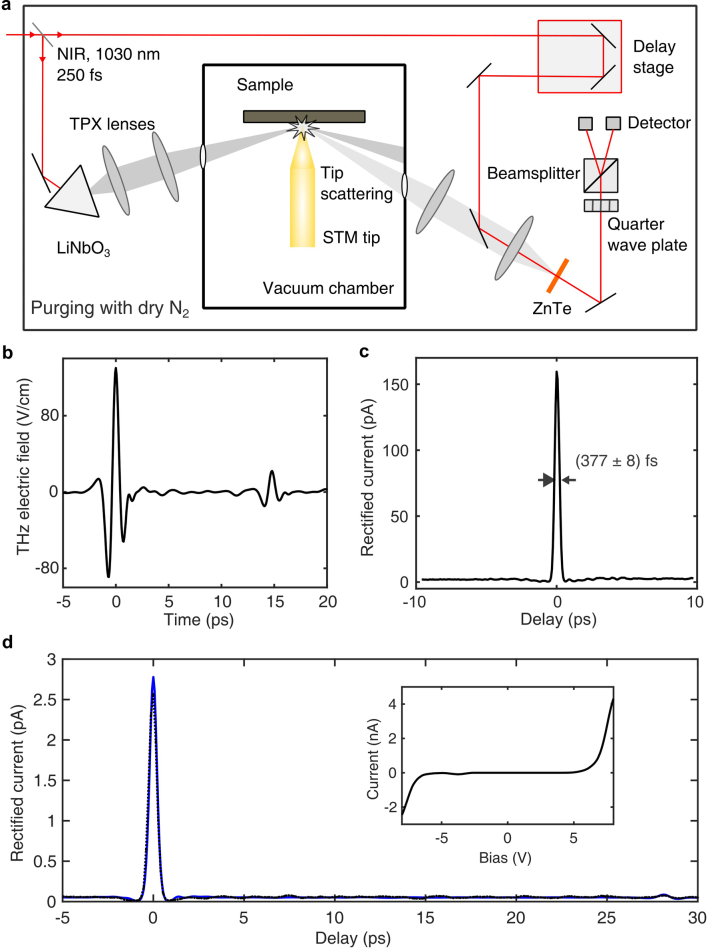
THz electric field waveform and THz pulse correlation on a Au(111) surface. **a**, Schematic of the THz electric field waveform characterization by electro-optic sampling of the THz light scattered from the STM tip. **b**, THz electric field waveform of the pump pulse measured by electro-optic sampling. The small peak at 15 ps stems from a reflection in the cryogenic window. **c**, Time trace of the THz-induced current recorded on Au(111) surface shows a full width at half maximum (FWHM) of 377 ± 8 fs. STM junction set point 130 pA at 22 mV. THz pulse pairs had a repetition rate of 10 MHz. **d**, Calculated pulse-pulse correlation signal (blue curve) using the measured THz electric field waveform in (b) and the I(V) curve measured on the Au(111) surface (inset). This THz electric field waveform can reconstruct the measured time trace (black dotted curve) well. Junction set point 10 pA at 100 mV. THz pulse pairs had a repetition rate of 1 MHz. The peak voltage induced by the excitation pulse was 3.8 V and that of the sensing pulse 3.0 V. Set point of I(V) curve −2.8 nA at −8 V. Source data

**Extended Data Fig. 2:**
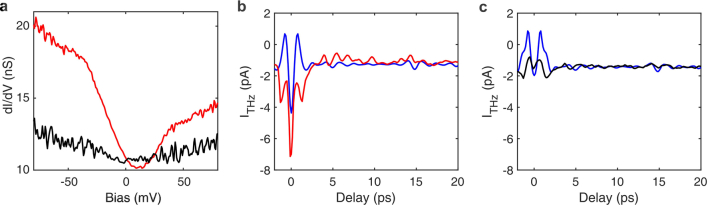
Differential conductance spectra and pulse correlation for 2H-NbSe_2_. **a**, Differential conductance spectra, dI/dV, of 2H-NbSe_2_ measured in the CDW phase at 20 K (red curve) and in the normal metal phase at 150 K (black curve). The dip observed in the range ± 35 mV is only present in the charge density wave phase (20 K) and absent in the normal metal phase (150 K). Tunnel junction set point for both spectra 1 nA at 80 mV, lock-in modulation frequency 784 Hz with amplitude 80 µV. **b**, Time trace of the THz-induced tunnel current at 20 K (red curve, same as Fig. 1d) and the calculated pulse-pulse correlation signal (blue curve) using a static dI/dV recorded at the same spot. Tunnel junction set point 1 nA at +1 mV. **c**, THz time trace at 150 K (black curve, same as Fig. 1f) and the calculated pulse-pulse correlation signal (blue curve) using a static dI/dV recorded at the same spot. Tunnel junction set point 2 nA at +1 mV. Source data

It is worth noting that the THz-induced tunnel current will contain not only the signal of the CDW dynamics but also a signal created by rectification of the electric field through the nonlinear *I*(*V*) characteristic of the tunnel junction^25–30^ and, in principle, by phonons of the NbSe_2_ surface^9,31^. Since the peaks at 0.45 THz and 1.1 THz appear in both the normal metal phase and the CDW phase, we attribute them to these CDW-unrelated sources. The contribution of the electric-field rectification results in a pulse correlation signal that can be calculated using the electric field waveform of the THz pulse and the *I*(*V*) characteristic of the tunnel junction^32–34^ (see Supplementary Section 1 for details). The THz pulse waveform at the STM tip is measured by electro-optic sampling of the THz light scattered from the tip (Extended Data Fig. 1b; see Methods for details). It shows minimal residual oscillation after the main pulse that peaks at 0.45 THz (Fig. 1b, dark blue). Hence, the pulse correlation signal accounts for the dip–peak structure at short delays in both the CDW and normal metal states, and also reproduces the reduction of the dip at zero delay at 150 K due to the more linear *I*(*V*) characteristic (Extended Data Fig. 2). However, the ultrafast decay and the low-frequency oscillatory signal are not created by the pulse correlation because they remain in the signal after subtracting the calculated pulse correlation from the measured signal (Supplementary Fig. 1a). The power spectral density (PSD) of the pulse correlation signal shows peaks at 0.5 THz and 0.6 THz but little spectral weight below 0.3 THz or above 0.6 THz (Supplementary Fig. 1b). This corroborates that the low-energy mode at 0.15 THz and the spectral weight between 0.6 THz and 0.9 THz are unique to the CDW phase.

CDWs exhibit collective oscillatory modes that are absent in the normal metal host crystal^1,9^. They are caused by excitations of the amplitude or phase of the order parameter. The amplitude mode excitation in NbSe_2_ was measured at 1.2 THz with Raman spectroscopy, but phase dynamics could not be resolved optically, yet^9,35^. As phase modes are expected at lower frequencies than the amplitude mode^1,36^, we posit that the sub-THz modes observed here stem from local phase excitations of the CDW state, detected locally by the tunnel current as conductance fluctuations. In the following, we verify this assignment by imaging the spatial structure of the low-frequency modes and comparing it with a numerical model of the CDW dynamics upon THz excitation in the STM junction.

**Fig. 2:**
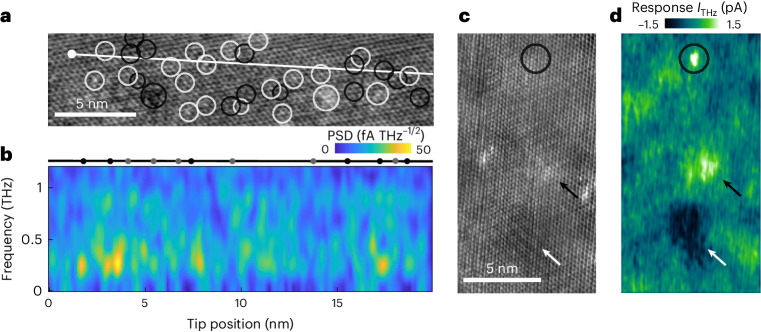
Atomically resolved CDW dynamics. **a**, Constant-current topography acquired in the CDW phase of 2H-NbSe_2_. Visible defects are highlighted by white circles (protrusions) and black circles (depressions). See Supplementary Fig. 2 for filtered images of CDW and defect signal. **b**, Spatially resolved PSD map as function of tip position and frequency obtained by FFT of a series of time traces measured along the white line shown in **a**. Junction set point 100 pA at 10 mV. FFT applies spatial Gaussian averaging with 0.3 nm width. The black line with dots is a guide to the eye indicating projected positions of defects relative to the scanned line (black, closer than 0.3 nm; grey, closer than 1 nm). **c**, Constant-current topography of a surface region with varying defect density (see Supplementary Fig. 3 for details). **d**, Map of the response of the THz-induced current to modulating the THz pulse amplitude recorded simultaneously with the topography (**c**). The THz response shows a positive signal in areas with high defect density (black arrow), a negative signal in areas with low defect density (white arrow) and an isolated defect with strong localized response (circle). Junction set point 1 nA at 1 mV. Source data

**Extended Data Fig. 3:**
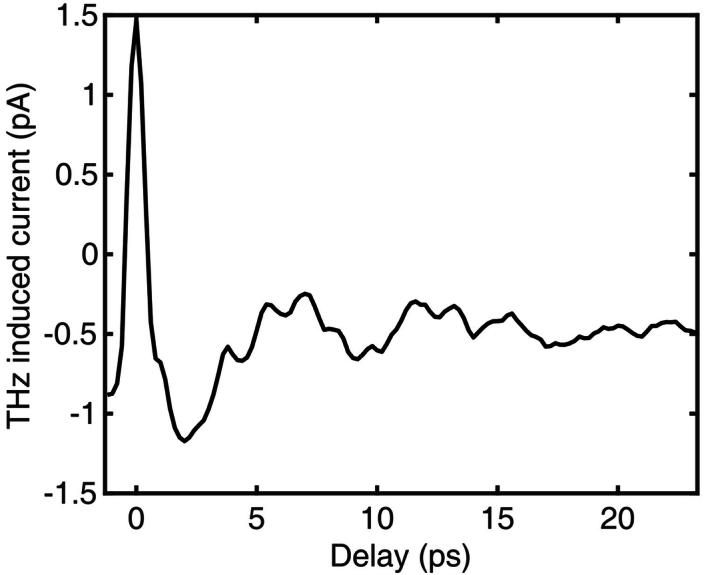
Ultrafast time trace of the THz-induced tunnel current recorded in a high-defect density region of 2H-NbSe_2_. The peak to positive THz-induced tunnel current at 0 ps delay suggests that the THz pulse transiently excites the CDW close to defects to a non-equilibrium state with a more symmetric I(V) characteristic. Temperature 20 K. Junction set point 4 nA at 1 mV. Source data

**Extended Data Fig. 4:**
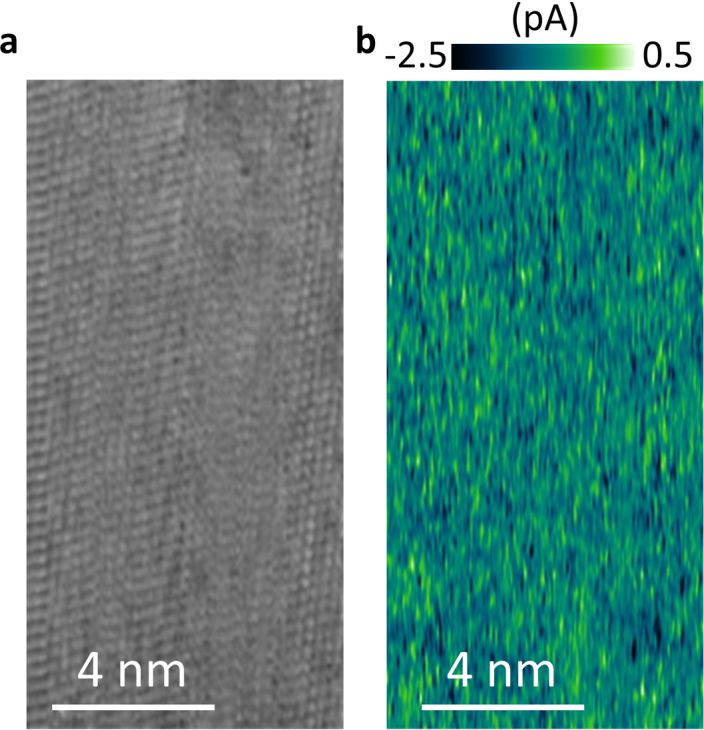
Spatially resolved THz-induced tunnel current in the normal metal phase of 2H-NbSe_2_. **a**, Constant-current topography recorded in the normal metal phase of 2H-NbSe_2_. Junction set point 1.5 nA at 1 mV. Temperature 141 K. **b**, Map of the response of the THz-induced current to modulating the THz pulse amplitude recorded simultaneously with the topography shown in (a). Source data

**Extended Data Fig. 5:**
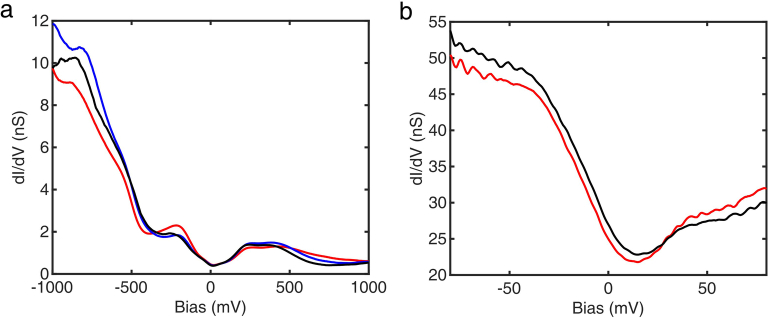
Spatially dependent conductance of 2H-NbSe_2_ in the CDW phase (20 K). **a**, Differential conductance, dI/dV, spectra recorded on different spots in Fig. 2c: dark defect (red curve), bright defect (blue curve), at 1 nm distance from a bright defect (black curve). Junction set point 914 pA at 1 V, lock-in modulation frequency 784 Hz with amplitude 10 mV. **b**, dI/dV spectra recorded at the same locations as the THz measurements shown in Fig. 4f, g: on the defect (black curve) and at 1 nm distance from the defect (red curve). Junction set point current 2 nA at 80 mV, lock-in modulation frequency 784 Hz with amplitude 80 µV. Source data

Phase excitations should be linked to pinning at defects; thus, we expect strong spatial variation in the measured response. Indeed, we find a direct correlation between defects visible in the topography (Fig. 2c) and the THz-induced tunnel current (Fig. 2d). With a continuous train of amplitude-modulated THz pulses sent to the tunnel junction while scanning the sample, we image where the NbSe_2_ surface responds strongly to the THz excitation (see Methods for details). At 20 K, in the CDW phase, regions with low defect density that show the pristine CDW have a negative signal (Fig. 2c,d, white arrow) matching the time trace shown in Fig. 1d, whereas areas with high defect density show a positive signal (Fig. 2c,d, black arrow) that relates to a peak in time traces recorded in those areas (Extended Data Fig. 3). We also find a small number of defects that have only little effect on the topography but show a strong localized response in the THz-induced current exceeding 1 pA (Fig. 2c,d, circle). By contrast, maps of the THz-induced current recorded at 141 K, in the normal metal phase, are markedly more uniform (Extended Data Fig. 4). This spatial structure cannot be solely attributed to variations in the static conductance of the sample because d*I*/d*V* spectra recorded on different defects and on the pristine CDW show only minor differences (Extended Data Fig. 5a) that cannot produce the highly heterogeneous THz response observed in the CDW phase (Fig. 2d).

The spatial structure of the phase excitations can be resolved by recording a series of PSDs along a line across a defect-rich region of the NbSe_2_ surface (Fig. 2a,b). The STM image shows many defects close to this line that appear either as localized depressions (dark) or protrusions (bright). The depressions have been attributed to Se vacancies and the protrusions to Nb intercalants below the top layer or other adatoms on the surface^17^. The spatially resolved PSD (Fig. 2b) shows that the sub-THz modes vary both in frequency and in amplitude across the sample on a length scale that is comparable to the average distance between defects in NbSe_2_ (refs. ^17,18,37^). The PSD signal below 0.5 THz is strongest in the region with higher defect density between 2 nm and 8 nm where it stays above the measurement noise floor over several unit cells of the CDW and is weaker in the region with low defect density between 9 nm and 16 nm. Peaks in the PSD at higher frequencies, between 0.6 THz and 0.8 THz, appear over shorter length scales down to 1 nm, for example, between 3 nm and 6 nm, 9 nm and 11 nm or 17 nm and 18 nm.

This strong spatial heterogeneity may explain why such low-frequency oscillatory modes have, thus far, not been observed by far-field optical spectroscopy. Spatial averaging would superimpose modes at many frequencies and either reduce the signal strongly or result in a featureless slow decay signal as has, for example, been observed for phase fluctuations in cuprates and nickelates^21,22^. It also highlights an intriguing dichotomy of charge-ordered states: dynamics that are associated with the state’s collective behaviour are intimately intertwined with localized interactions with defects, leading to a highly heterogeneous ultrafast response.

**Fig. 3:**
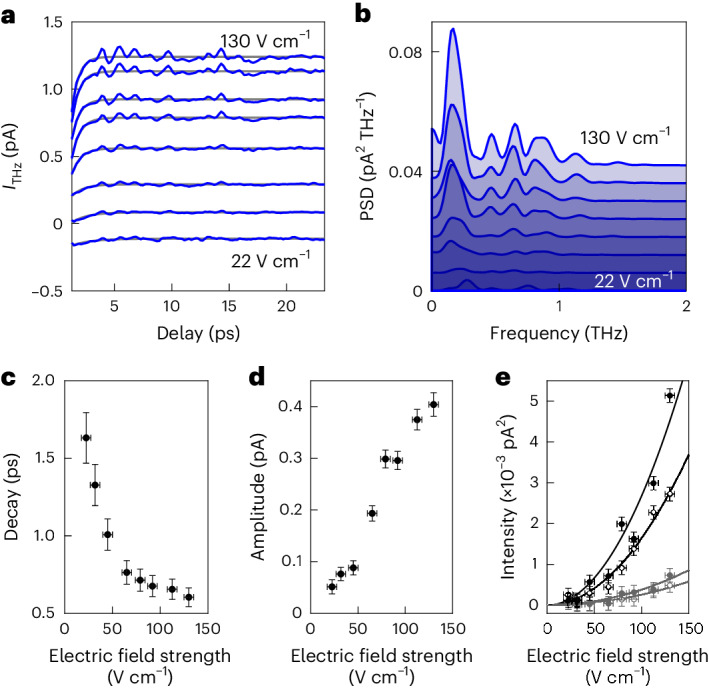
THz-induced local excitation of CDW dynamics. **a**, Dependence of time traces of the THz-induced tunnel current on electric field strength of the excitation pulse (blue lines) and exponential fits (grey lines). Electric field strength increases from bottom to top. The curves are vertically shifted for clarity. Junction set point 1 nA at 1 mV. **b**, PSD of the time traces in **a**. The curves are shifted vertically, and areas under them are shaded for clarity. **c**,**d**, Decay constant (**c**) and amplitude (**d**) of the exponential decay as a function of excitation pulse strength extracted from the time traces in **a** (see Methods for fit details). **e**, Intensities of the peaks in the PSD in **b** as a function of excitation pulse strength for the CDW-related signal at 0.16 THz (black dots) and 0.6–0.9 THz (open black circles), and the CDW-unrelated signal at 0.45 THz (grey dots) and 1.1 THz (open grey circles). Solid curves are a guide to the eye assuming quadratic scaling. The horizontal error bars in **c**–**e** represent uncertainties of the measured THz electric field. The vertical error bars in **c** and **d** correspond to 0.65 confidence level derived from the fits in **a**. The vertical error bars in **e** are standard deviations within the selected frequency ranges. Source data

To understand how these localized CDW dynamics are excited, we focus on the ultrafast decay observed within the first few picoseconds after the THz excitation pulse and investigate its dependence on the electric field strength of the THz pulses (Fig. 3a). By fitting the decay after the pulse–pulse correlation signal in the measured time traces (Fig. 3d), we find that the decay amplitude increases approximately linearly from 0.05 pA to 0.41 pA with increasing field strength, indicating that the excitation is driven by the electric field of the THz pulse. The decay time constant decreases nonlinearly from 1.7 ± 0.2 ps to 0.6 ± 0.1 ps (Fig. 3c). A similar decay is observed in the normal metal phase (Fig. 1f). This behaviour matches to transient reflectivity measurements of NbSe_2_ that found decreasing electron–phonon relaxation times with increasing laser fluence^23^ and is consistent with an electron bath thermal relaxation after the pump pulse^23,38,39^. This points to an excitation mechanism in which the THz electric field acts on the mobile charge carriers of the NbSe_2_ sample and introduces ultrafast heating of the electron system both in the normal metal and CDW phase.

**Extended Data Fig. 6:**
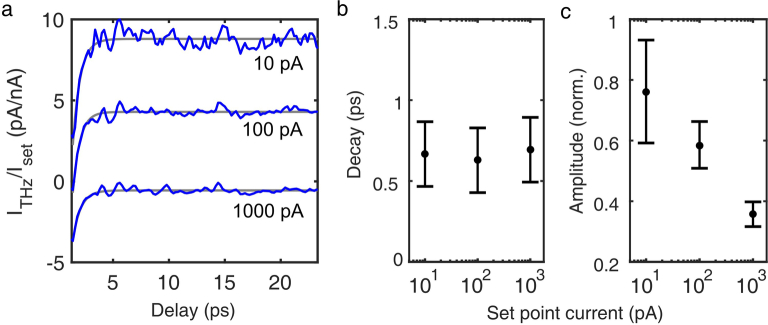
Set point dependence of the excitation of CDW dynamics. **a**, Time traces of the THz-induced current for increasing junction set point current at constant bias, 1 mV, (blue curves) and exponential fits (grey lines). The curves are normalized to the set point current, *I*_*set*_, to remove the influence of the increasing tunnel junction conductance on the tunneling probability during the sensing pulse. Spectra are shifted vertically for clarity. **b, c**, Decay constant (b) and amplitude (c) of the exponential fits shown in (a) as a function of set point current. Error bars in (b) and (c) correspond to 0.65 confidence level derived from the fit. Source data

By retracting the STM tip 2.1 Å from the surface (Extended Data Fig. 6a), the tunnelling probability is reduced by a factor of 100 (confirmed by applying a constant voltage and observing the reduction in the direct tunnel current). Despite this, we find that the time constant of the ultrafast decay remains constant at 0.7 ± 0.2 ps (Extended Data Fig. 6b) and its relative amplitude decreases only by a factor of 2 (Extended Data Fig. 6c). This does not match to an excitation by tunnelling electrons but is consistent with the electric-field dependence shown in Fig. 3, further corroborating that the CDW excitation under the STM tip is directly driven by the electric field of the THz pulses.

Consistent with this, we observe that the PSD of the CDW-related modes at 0.16 THz and between 0.6 THz and 0.9 THz increases approximately quadratically upon increasing the excitation pulse’s electric field strength (Fig. 3e).

The CDW-unrelated peaks at 0.45 THz and 1.1 THz remain much weaker, reaching only between 10% and 20% of the CDW-related peaks for 130 V cm^−1^ (electric field strength measured in the THz far field), but appear to scale nonlinearly with excitation field strength as well. This suggests that they originate from other processes excited by the THz electric field in the STM junction, such as acoustic phonons, which are launched by the ultrafast Coulomb force that scales quadratically with THz field strength^40^. Despite similar scaling with electric field, CDW excitations and phonons can be differentiated, in particular by their behaviour across the CDW phase transition, where we found the 0.16 THz and 0.6–0.9 THz modes to vanish in the normal metal phase at high temperature (Fig. 1g).

**Extended Data Fig. 7:**
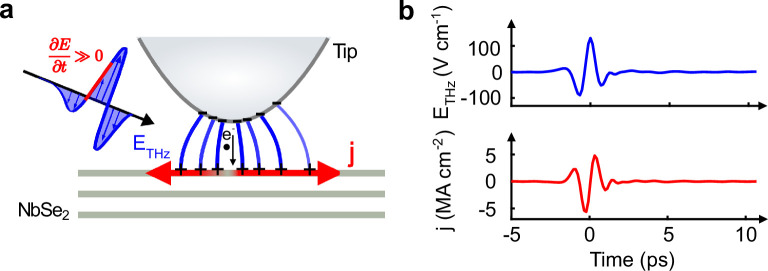
Screening current on the sample surface induced by THz electric field. **a**, Surface charges at the tip apex (depicted as −) and at the sample surface (depicted as +) cause the enhancement of the THz electric field in the tunnel junction, *E*_*THz*_, (blue curves). The electric field oscillates at 0.55 THz with large changes of the electric field, *∂E*/*∂t*, that result in large a.c. screening current, on the NbSe_2_ sample surface (red arrows). The black arrow symbolizes the tunneling electrons between tip and sample. **b**, THz electric field (blue curve) and induced screening current density, *j*, on the sample surface (red curve) in the vicinity of the tip apex (see Supplementary text 2 for details on calculation). Source data

For the field strength of 130 V cm^−1^ that our THz source achieves in the optical focus, the enhanced field in the tunnel junction exceeds 1 MV cm^−1^ (refs. ^32,41^). Whereas the application of a static electric field of this magnitude in the STM does not affect the CDW, the oscillating electric field of the THz pulse induces a tremendous time-dependent screening current^27,40^ at the surface. The THz field oscillates at a centre frequency of 0.55 THz. The resulting screening current is on the order of 10^6^ A cm^−^^2^ within a few nanometres from the tip apex (Extended Data Fig. 7). The concomitant Joule heating quickly raises the effective electron temperature, which can drive thermal phase transitions at ultrafast time scales^42,43^. Transport measurements of mesoscopic samples have observed that strong in-plane currents are sufficient to cause phase transitions in 2D commensurate CDW materials^44^ and to induce MHz-range fluctuations from field-induced phase motion of the 1D CDW state of NbSe_3_ (ref. ^14^). Hence, the THz-induced screening current can drive the CDW into non-equilibrium states. Both the thermal relaxation and the phase fluctuations by which this state relaxes modulate the occupation and the density of states close to the Fermi energy^1,21,45^ on a picosecond time scale. The time-delayed sensing pulse picks up this modulation locally by THz-induced electron tunnelling^28–30^.

**Fig. 4:**
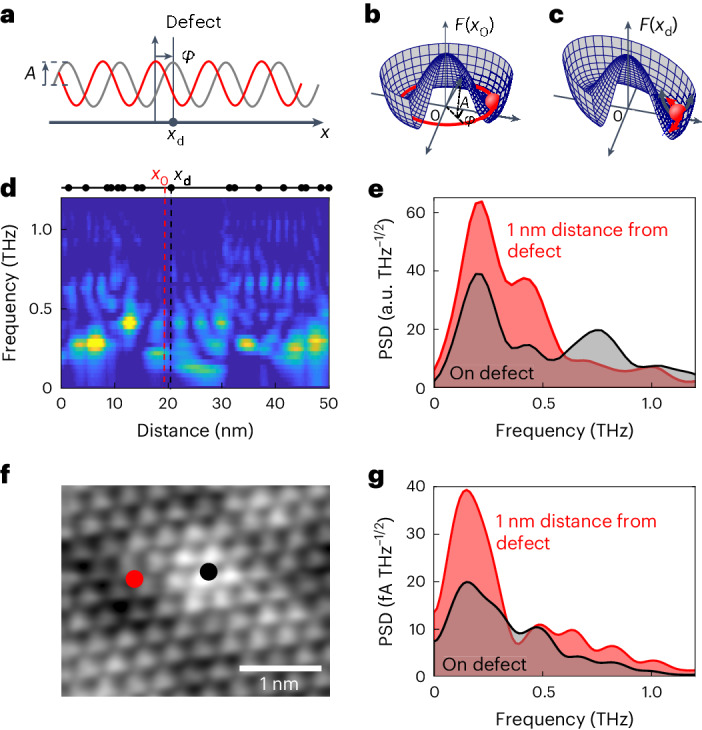
Model of ultrafast phase motion in a CDW. **a**, Sketch of the charge distribution of a CDW characterized by its amplitude, *A*, and phase, *φ*, without (red curve) and with (grey curve) pinning at a defect (black dot). **b**,**c**, Sketch of the local free energy, *F*(*x*), showing the effect of pinning on the defect (**c**), *F*(*x*_d_), compared with the undisturbed surface (**b**), *F*(*x*_0_). **d**, Spatially resolved PSD map of the CDW phase dynamics resulting from a random impurity distribution indicated by the black dots. Colour scale from low (blue) to high (yellow). See Supplementary Section 3 for details of model. **e**, PSDs of the simulated time traces on a single defect (black curve) and at 1 nm distance from the defect (red curve). **f**, Measured constant-current topography of a defect in 2H-NbSe_2_ located at a CDW maximum. Junction set point 28 pA at 6 mV. **g**, PSDs of time traces measured on the atomic defect shown in **f** (black curve) and at 1 nm distance from the defect (red curve). Locations of the measurements are marked in the STM image (**f**) by a black dot (on defect) and a red dot (1 nm distance). Junction set point 1 nA at 1 mV. Differential conductance spectra, d*I*/d*V*(*V*), measured at the same positions are plotted in Extended Data Fig. 5b. Areas under curves in **e** and **g** are shaded for clarity. Source data

To verify how the THz-induced localized screening current excites CDW dynamics, we model the response of the CDW with an extended Ginzburg–Landau model^46,47^ that uses a time- and position-dependent free energy density (Fig. 4b,c; see Supplementary Section 3 for details). By assuming a Drude-like conductivity, the screening current of the excitation pulse can be described as a damped acceleration of the CDW at the NbSe_2_ surface and concomitant Joule heating that affects the CDW’s free energy. The impact of atomic-scale defects is included by a pinning potential landscape with localized and randomly distributed potentials that locally favour a specific phase and distort the free-energy density accordingly (Fig. 4a,c). The simulated random defect distribution matches the defect density of the sample, but the exact pinning potential landscape of the sample cannot be reproduced quantitatively. Thus, the model highlights the emergence of low-frequency CDW dynamics in disordered pinning potential landscapes but does not attempt quantitative fitting of the measurement.

The model shows that the THz-induced screening current in the NbSe_2_ surface can, indeed, excite local dynamics of the CDW. We find that these dynamics are predominantly oscillations of the CDW phase in the pinning potential landscape of the defects (Fig. 4d). Amplitude oscillations are negligible as they decay quickly^23^ and our THz pulse lacks bandwidth to efficiently excite them.

Furthermore, the model indicates that the phase dynamics consist of a complex spatially heterogeneous pattern in frequency and amplitude that extends from 0.1 THz to just above 1.1 THz. Strong phase excitations that extend spatially for up to 5 nm appear between 0.2 THz and 0.5 THz in areas with high defect density, whereas regions with low defect density feature weaker excitations that extend to lower frequency. Above 0.7 THz, the phase excitations become more localized, with substantial intensity appearing only in confined regions. Thus, the phase dynamics in the random, atomically localized pinning potential landscape qualitatively reproduce the experimentally observed trend in the spatially resolved PSD that features a stronger response below 0.5 THz in the vicinity of defect-rich areas and more localized signals at higher frequencies (Fig. 2d).

This behaviour can be understood by the time-dependent motion of the CDW close to a single atomic impurity. The THz pulse accelerates the CDW phase on the entire surface and causes a temporary reduction of the CDW amplitude through the Joule heating of the electron system. As the impurity breaks translational symmetry, this results in the build-up of a phase distortion at the impurity during the pulse. After the pulse, the distortion relaxes by phase excitations that propagate away from the impurity. Similar phase excitations launch from each defect within the few-nanometre reach of the THz-induced screening current. They overlap in areas between the defects, thus creating the observed spatially heterogeneous pattern of sub-THz modes.

Importantly, the model indicates that these modes are suppressed directly at a phase-pinning impurity and appear in its immediate vicinity (Fig. 4e), which we can confirm experimentally by local measurement with the STM at a prominent defect on the surface (Fig. 4f). We find that the spectral weight at 0.15 THz and between 0.6 THz and 0.8 THz reduces by more than a factor 2 when directly above the defect compared with a point just 1 nm away from it (Fig. 4g), in good qualitative agreement with the calculated behaviour. By fitting the model to reproduce phase excitations in the same 0.15–0.8 THz frequency band as observed in the experiment (see Supplementary Table 1 for model parameters), we obtain a group velocity for the phase excitations of 3.2 nm ps^−1^ (see Supplementary Section 3.2 for details), which is consistent with the theoretically predicted velocity of phasons in CDWs^1^.

We conclude that the atomic spatial resolution of the STM-based THz spectroscopy described here locally detects charge density dynamics in NbSe_2_. After excitation by a THz pulse, the CDW state relaxes through low-energy phase excitations in the sub-THz range that are enabled by atomic-sized defects that pin the CDW and are heterogeneous on the nanometre scale.

Locally imaging collective charge order dynamics may also elucidate the impact of spatially structured fluctuations on phase competition between charge order and other phenomena such as superconductivity or complex magnetism^11^. We emphasize that the electric-field-driven excitation mechanism we have described here should be capable of exciting nanoscale dynamics in a larger class of layered materials and may enable imaging of phase boundary motion in metal-to-insulator transitions, localized fluctuations in 2D magnets or even pair-breaking dynamics in superconductors.

## Methods

### Experimental setup

The 2H-NbSe_2_ crystals are cleaved in ultrahigh vacuum (with a base pressure of <2 × 10^−10^ mbar) at room temperature using Kapton tape and inserted into the scanning tunnelling microscope head immediately. The sample is cooled down to 150 K and 20 K, which is above and below the CDW transition temperature (*T*_CDW_ = 33 K)^24^, respectively. Ground and electro-polished commercial Platinum-iridium tips are used. Before measurements on the 2H-NbSe_2_ sample, the tips are repeatedly dipped a few nanometres into a Au(111) surface until they show stable THz signal and a flat d*I*/d*V* spectrum.

THz pump–probe measurements are performed in a home-built low-temperature STM designed to efficiently couple the THz pulses onto the STM tip. A detailed description of the setup can be found in ref. ^32^. The THz pulse pairs are generated via optical rectification of 250 fs near-infrared (NIR) pulses (1,032 nm) using a lithium niobate (LiNbO_3_) crystal (Extended Data Fig. 1a). A tilted pulse front method is used to increase the efficiency of the THz generation. All measurements use a repetition rate of 41 MHz. The whole THz beam path is purged with dry N_2_. The THz beam is focused onto the STM tip directly above the tunnel junction through a *z*-cut quartz vacuum window using a pair of polymethylpentene lenses.

### THz electric field in the STM tunnel junction

The THz light scattered from the STM tip is collected through a second *z*-cut quartz vacuum window using polymethylpentene lenses in a geometry analogous to light detection in scattering near-field optical microscopy^48,49^ so that the electric field waveform of the scattered THz light can be measured by electro-optic sampling using a ZnTe crystal. It contains near-field information of the THz pulse at the tunnel junction and all pulse distortions such as reflections at cryogenic windows that occurred in the THz beam path up to the STM tip (Extended Data Fig. 1b) and accurately reproduces pulse–pulse correlation measurements on a Au(111) surface (Extended Data Fig. 1d). The measured electric-field waveform verifies that the THz pulses used in this experiment consist of a single optical cycle and have a spectral width that stretches beyond 1 THz (Fig. 1b and Supplementary Section 1).

The strong field enhancement at the tip apex amplifies the electric field component of the THz pulses, resulting in an extremely strong electric field in excess of 1 MV cm^−1^ under the STM apex. This also results in a transient voltage pulse in the tunnel junction that causes tunnelling of electrons leading to a THz-induced tunnel current^25,32,41^. At the chosen repetition rate of 41 MHz, the THz pulses have a far-field peak electric field strength of 130 V cm^−1^ that induces a peak pulse voltage of 0.1 V in the tunnel junction (estimated by fitting the THz-induced tunnel current to the instantaneous response calculated from the static d*I*/d*V* spectra recorded at 20 K and 150 K; Extended Data Fig. 2, as described in ref. ^32^). This voltage pulse amplitude matches to the energy scale of the CDW-induced dip^6^ observed in the d*I*/d*V* spectrum at 20 K (Extended Data Fig. 2). The central positive THz electric field peak induces electron tunnelling from sample to tip, which corresponds to a negative current and a negative THz-induced voltage in the common convention of sample bias. All measurements on NbSe_2_ use constant THz pulse amplitude, except the measurement of the electric-field dependence of *I*_THz_ (Fig. 3a), where the amplitude of the excitation pulse is adjusted with an acousto-optic modulator while keeping the sensing pulse amplitude constant.

### Ultrafast time traces of the THz-induced tunnel current

Ultrafast time traces of the THz-induced tunnel current are recorded using pump–probe methodology: a 50:50 beam splitter creates pulse pairs in the NIR beam path, and acousto-optic modulators adjust each pulse’s amplitude. A motorized linear stage placed in the optical path of one pulse controllably sweeps the time delay between the two pulses. These pulse pairs are converted to THz pulses in the lithium niobate crystal. All measurements use a step size of 30 µm corresponding to time steps of 0.2 ps. The time axis for all measurements is set to *τ* = 0 ps when both pulses coincide at the STM, and we swept the delay time from −2 ps up to +23.3 ps. This delay time interval includes the pulse–pulse correlation signal created by the overlap of the THz pulses at zero delay and covers the range in which the oscillatory dynamics occur.

The first pulse of each pulse pair can excite the sample, and the trailing pulse measures the resulting ultrafast modulation of the tunnel junction conductivity. We use lock-in detection to separate the tunnel current contributions of the pulses: a mechanical chopper modulates one pulse on and off at a frequency of 617 Hz, and the resulting tunnel current variation at this frequency is isolated with a lock-in amplifier. At positive delay times, *τ* *>* 0 ps, the chopped pulse follows the non-chopped pulse and is therefore sensitive to the ultrafast dynamics excited by the non-chopped pulse. Hence, we termed the chopped pulse ‘sensing pulse’ and the non-chopped pulse ‘excitation pulse’.

The STM feedback loop is kept off and the d.c. bias voltage is set to 0 V during all time trace measurements. Then, the lock-in-detected tunnel current originates solely from the THz-induced tunnel current of the sensing pulse, *I*_THz_. The time traces are recorded by slowly sweeping the time delay between excitation and sensing pulse while recording *I*_THz_. Delay-time-dependent variations of *I*_THz_(*τ*) include ultrafast dynamics.

### Map of the THz-induced tunnel current

To record a spatial map of the surface’s THz response, we slowly scan the STM tip across the surface while exciting the tunnel junction with amplitude-modulated THz pulses. A small d.c. bias voltage is applied and the feedback loop is active to stabilize the tip height for the long duration of the scan. The THz amplitude modulation is achieved by setting the pulse-pair delay to 300 fs and chopping one pulse on and off at 617 Hz. At this delay, the THz pulses overlap, increasing the THz electric field amplitude when both pulses are on, but the NIR pulses do not overlap, avoiding possible interference in the lithium niobate. The resulting modulation of the THz-induced tunnel current is isolated from the d.c. tunnel current used for STM feedback by a lock-in amplifier so that topography and map of the THz-induced tunnel current can be recorded simultaneously.

### Power spectral density

PSDs are calculated from the oscillatory part of the time traces by fast Fourier transform (FFT) (Fig. 1b, delay range 3.0–20.0 ps; Figs. 1g, 2b, 3b and 4e,g and Extended Data Fig. 3b, delay range 3.6–23.3 ps). The regions of short delay times are excluded from the FFT to avoid spurious signals in the PSD from the ultrafast decay signal and pulse–pulse correlation signal. Before FFT, we subtract the average of the signal and apply a moving Hann window function. This avoids a 0 THz peak and otherwise possible windowing artefacts in the PSD. The window width is 12 ps for Figs. 1b and 4e,g, 15 ps for Figs. 1g and 3b and 7 ps for Fig. 2b.

### Ultrafast relaxation fit

The ultrafast decay observed in the time traces for short delay times is fitted with a single exponential function of the form ${I}_{{\mathrm{d}}}\exp \left(-\frac{\tau }{{\tau }_{{\mathrm{d}}}}\right)+{I}_{0}$, where *I*_d_ is the amplitude of the decay, *I*_0_ is the signal baseline at long delays and *τ*_d_ is the time constant of the decay. Delay times shorter than 1.6 ps are excluded from the fit to avoid the pulse correlation signal created by the overlapping excitation and sensing pulse from skewing fit results. Fits were repeated after subtracting the calculated pulse correlation signal from the measured time trace and show the same decay constants within the fit uncertainty (Supplementary Figs. 1 and 4).

## Online content

Any methods, additional references, Nature Portfolio reporting summaries, source data, extended data, supplementary information, acknowledgements, peer review information; details of author contributions and competing interests; and statements of data and code availability are available at 10.1038/s41567-024-02552-7.

## Supplementary information

Supplementary Information Supplementary text, Table 1, Figs. 1–4 and references.

## Source data

Source Data Fig. 1 Source data for Fig. 1b–g.

Source Data Fig. 2 Source data for Fig. 2a–d.

Source Data Fig. 3 Source data for Fig. 3a–e.

Source Data Fig. 4 Source data for Fig. 4d–g.

Source Data Extended Data Fig. 1 Source data for Extended Data Fig. 1b–d.

Source Data Extended Data Fig. 2 Source data for Extended Data Fig. 2a–c.

Source Data Extended Data Fig. 3 Source data for Extended Data Fig. 3.

Source Data Extended Data Fig. 4 Source data for Extended Data Fig. 4a,b.

Source Data Extended Data Fig. 5 Source data for Extended Data Fig. 5a,b.

Source Data Extended Data Fig. 6 Source data for Extended Data Fig. 6a–c.

Source Data Extended Data Fig. 7 Source data for Extended Data Fig. 7b.

## Data Availability

Details on the calculation of the time-dependent screening current and the CDW dynamics are included in Supplementary Sections 2 and 3. More data are available from the corresponding author upon reasonable request. Source data are provided with this paper.

## Code availability

Code is available from the corresponding author upon reasonable request.
