# Supplementary material for: Terahertz spectroscopy of collective charge density wave dynamics at the atomic scale
Source: Nat Phys. 2024 Jul 15;20(10):1603–8. doi: 10.1038/s41567-024-02552-7 (PMC11473363; doi:10.1038/s41567-024-02552-7)
Supplement: Supplementary file 1 — Supplementary text, Table 1, Figs. 1–4 and references. [file 41567_2024_2552_MOESM1_ESM.pdf]

nature physics

Supplementary information

https://doi.org/10.1038/s41567-024-02552-7

Terahertz spectroscopy of collective charge density wave dynamics at the atomic scale

In the format provided by the authors and unedited

Table-of-contents:

Supplementary Text
Supplementary Table S1
Supplementary Figures S1 to S4
Supplementary References

Supplementary Text

1. THz pulse characterization

To characterize the electric field waveform of the THz pulse in the STM junction, we collect the scattered THz light from the STM tip apex by a pair of TPX lenses and image it into a ZnTe crystal, Extended Data Fig. 1a. The collection optics is set up such that the specular reflection of THz light from the sample is not measured. Then, we use a 250 fs NIR pulse from the pump laser to measure the time-dependent electric field by electro-optic sampling in the ZnTe crystal, Extended Data Fig. 1b. The scattered light from the tip contains near-field information from the STM tip as well as all distortions that occurred in the THz beam path and better reconstructs the THz-induced current measured in the STM[1,2].

In addition, several measures minimize distortion of the THz waveform: the window material used for vacuum chamber and cryogenic shields are z-cut quartz, which has a uniform transmission over the full bandwidth of our THz pulses[3]; all focusing optics are manufactured from TPX polymer that also has flat transmission in the bandwidth of our THz pulse[4]; the entire beam path outside the vacuum chamber is purged with dry nitrogen to reduce the tail of the THz waveform that is otherwise present due to THz absorption by water in the air[5].

We use a Au(111) crystal mounted on the same sample holder as the $NbSe_2$ sample to verify that the waveform detected by electro-optical sampling matches to the transient THz-induced voltage in the STM junction. We sweep one THz pulse (termed sensing pulse in the main text) across the other (termed excitation pulse in the main text) and detect the THz-induced tunnel current by the same lock-in measurement as described in the Methods section of the main text. We assume that Au(111) contains no dynamics in the bandwidth of the THz pulse, so that the signal is entirely created by the instantaneous tunneling response to the time-dependent electric field. Then, this measurement represents a pulse-pulse correlation measurement. The signal can be calculated from the time-dependent electric field and the measured I(V) characteristic of the Au(111) surface. This method is described in refs.[6–8] and will be summarized in the following.

The THz-induced tunnel current in the pulse-pulse correlation can be calculated as:

$$\bar{I}(\tau) = \int \sigma(V_{THz}(t,\tau)) \cdot V_{THz}(t,\tau)dt$$

where $\sigma(V)$ is the voltage-dependent conductance of the tunnel junction that is measured by static dI/dV spectroscopy (Extended Data Fig. 2a), and V_{THz} is the transient bias voltage induced by the THz pulse. Here, we assume that V_{THz} is proportional to the waveform of the electro-optic sampling signal of the tip-scattered excitation and sensing pulse, $E_{THz}^{e,s}$, with a proportionality constant α. This results in the following relation between V_{THz} and E_{THz}.

$$V_{THz}(t,\tau) = V_{THz}^{e}(t,\tau) + V_{THz}^{s}(t) = \alpha\left(E_{THz}^{s}(t-\tau) + E_{THz}^{e}(t)\right).$$

Since $\sigma(V)$ is experimentally determined by dI/dV spectroscopy, the function $\bar{I}(\tau)$ has only one free fit parameter, α, which accounts for the field enhancement and the relation between electric field and voltage in the tunnel junction of the STM. We determine α by matching $\bar{I}(\tau)$ to the average of the measured THz-induced tunnel current at long delay times with $\tau > 10$ ps. Then, the peak structure at short delays, where the pump and probe pulses overlap, is obtained without free fit parameters.

We find that the pulse-pulse correlation calculated using this method faithfully reproduces the measured pulse-pulse correlation signal on Au(111) and fits the measured THz-induced current in the tunneling regime quantitatively, Extended Data Fig. 1d. This verifies that the electric field waveform detected by electro-optic sampling of the tip-scattered THz pulses corresponds well to that in the STM tunneling junction.

Due to the good match on Au(111) we use this THz waveform to calculate the pulse-pulse correlation signal expected on $NbSe_2$. In contrast to Au(111), the measurements on $NbSe_2$ differ significantly from the calculated pulse-pulse correlation, indicating the presence of ultrafast dynamics in the measured signal.

The measured THz waveform (Extended Data Fig. 1b) consists of a single optical cycle with very minimal waveform tail for delay times larger than 1.6 ps. A pulse replica that has 16 % amplitude compared to the main pulse is present at 15 ps. It stems from a pulse reflection in the quartz windows at the cryogenic shield. To prevent this pulse repetition from distorting the power-spectral density plots shown in Fig. 1-4 and Fig. S1 we ensure that the main pulse and its echo are never included together in the FFT calculation. For the calculation of the PSD of the main THz pulse (Fig. 1b, light blue curve), we exclude delay times larger than 3 ps. And for the PSD of the THz waveform tail (Fig. 1b, blue curve) and for the PSD of the low-frequency phase oscillations we exclude delay times shorter than 3.6 ps.

The THz pulses used in this experiment have a central frequency of 0.55 THz and a relevant bandwidth of just over 1 THz. They consist of a single optical cycle with a very minimal waveform tail past the main pulse. The main peak in the electric field waveform has a falling edge of 0.4 ps (measured as the time for the 90% to 10% transition of the electric field). These pulses produce a pulse correlation signal on Au(111) with a full width at half maximum (FWHM) of 377 ± 8 fs when the pulse amplitude is increased such that the THz pulses can reach the strongly nonlinear region of the Au(111) I(V) characteristic that efficiently rectifies the THz pulses, Extended Data Fig, 1c. Upon reducing the THz pulse amplitude, the rectification becomes less efficient and the THz-induced tunnel current reduces to a few picoamperes. Despite this, the shape and width of the measured pulse correlation is well reproduced using the above-described calculation, Extended Data Fig. 1d. This indicates that the ultrafast tunnel current during a THz pulse follows the equation $I(t) = \sigma(V_{THz}(t)) \cdot V_{THz}(t)$, and allows an estimate of the effective time resolution of the ultrafast time traces by the FWHM of $I(t)$, and we find this effective time resolution to be 0.4 ps or better.

2. Screening current calculation

THz pulses focused onto the tunnel junction of a STM induce a strong transient electric field between tip and sample that is highly divergent. This is a consequence of the tip-enhancement of the electric field at the tip apex and caused by the induction of transient surface charges on the tip and sample[9]. The charge distribution on the sample surface, $\sigma(\vec{r},t)$ with $\vec{r} = (x, y, z = 0)$ depends on the profile of the electric field $E_{THz}(\vec{r},t)$ between tip and sample, Extended Data Fig. 7. Since the THz electric field oscillates, the charge distribution must also oscillate in time. This leads to a strong alternating screening current $j(\vec{r},t)$ on the sample surface. Because the inter-layer resistivity in $NbSe_2$ is much larger than the in-plane resistivity[10], this

screening current must have predominantly in-plane character. Hence, it can be carried either by the residual free electrons or by phase motion of the CDW in the $NbSe_2$ layers.

To estimate the magnitude of the screening current, we assume that the THz electric field is completely screened at all times within the first surface layer of 2H-$NbSe_2$. Then, the induced charges are also confined to the surface layer and are given by the surface charge distribution $\varrho(\vec{r},t) = \sigma(\vec{r},t)\delta(z)$ which can be calculated at every time *t* using the Poisson equation

$$\vec{\nabla}^2\varphi(\vec{r},t) = \frac{\varrho(\vec{r},t)}{\varepsilon_0} \tag{1}$$

where ε_0 is the vacuum permittivity and $\varphi(\vec{r},t)$ is the potential difference between tip and sample. Under the assumption of screening in the first $NbSe_2$ layer, this potential difference is equal to the THz-induced voltage that was determined experimentally. To solve the Poisson equation, we approximate the tunnel junction with a sphere-cone tip geometry and use an image charge model[11]. The screening current can be calculated directly from the charge distribution by the continuity equation

$$\frac{d\varrho(\vec{r},t)}{dt} + \vec{\nabla}\cdot j(\vec{r},t) = 0. \tag{2}$$

For the measured peak THz-induced voltage of 0.1 V and at a center frequency of 0.55 THz, this calculation shows that the screening current is extremely high. Surface current densities as high as $0.4\ A\cdot cm^{-1}$ are reached in the vicinity of the STM tip, during the central part of the THz pulse, when the rate of change of the electric field, $\partial E/dt$, is maximal. This current magnitude is comparable to THz-induced surface current densities observed on other layered materials with time-resolved angular photoemission spectroscopy[12]. Considering the thickness of one 2H-$NbSe_2$ layer, $0.6\ nm$[13], this corresponds to a current density of $7\times10^6\ A\cdot cm^{-2}$, Extended Data Fig. 7, which can be expected to lead to a strong excitation of the CDW[14].

3. Modelling CDW dynamics using Ginzburg Landau theory and Euler Lagrange formalism.

Ginzburg Landau theory has proven effective to describe the temperature-dependent phase transition of the CDW state to the normal metal states[15], the appearance of amplitude oscillations upon ultrafast laser excitation[16,17], as well as DC and AC electronic transport using in-plane electric fields[18,19]. Such Ginzburg Landau theory has recently been used to also describe the appearance of topological defects in stationary CDW states[20].

Here, we extend the Ginzburg Landau model to account for spatially dependent amplitude and phase dynamics of the CDW in one dimension. We describe the charge density in the CDW state as a perturbation of the equilibrium charge density $\rho_0(x)$ of the system in the normal metal state. Then, the total charge density with CDW is $\rho(x) \equiv \rho_0(x) - \alpha(x)$, and the CDW is fully characterized by $\alpha(x)$ which we define as

$$\alpha(x) \equiv A(x)\cos\big(q_0x + \varphi(x)\big) \tag{3}$$

where $A(x)$ is the spatially dependent charge amplitude, $\varphi(x)$ the spatially dependent phase and q_0 the ideal 1D wave number. $\alpha(x)$ serves as the complex order parameter of the system.

For a CDW that allows spatial variations of the order parameter, we can then extend the typically used free energy functional into a free energy density $\mathcal{F}$ of the form

$$\mathcal{F} = \underbrace{aA^2 + bA^4 + c_A(\partial_x A)^2}_{\mathcal{F}_A} + \underbrace{c_\varphi A^2(\partial_x\varphi)^2}_{\mathcal{F}_\varphi} + \underbrace{\alpha U}_{\mathcal{F}_U} \tag{4}$$

where $\mathcal{F}_A$(x), $\mathcal{F}_\varphi$(x) and $\mathcal{F}_U$(x) are the amplitude, phase and impurity energy densities. The parameters, a, b, c_A and c_φ parametrize the shape of the free energy functional and we consider them to not depend on position. The term $\mathcal{F}_A$(x) describes the phase transition into the CDW phase, such that the temperature dependence of the parameter $a(T) = \tilde{a} \cdot (T - T_c)$ shifts the free energy density minimum in $|A|$ from zero to a finite value of $|A|$ below the critical temperature, T_C,[21]. The derivate term in $\mathcal{F}_A$ introduces an energy cost to spatial variations of the amplitude of the order parameter, and the term $\mathcal{F}_\varphi$(x) attributes an energy cost to spatial variations of the CDW phase at finite CDW amplitudes. This is motivated by previous observations of spatially uniform CDW order in materials with low defect densities[22,23].

The interaction of the CDW with defects is treated as a charge-dependent interaction with local pinning potentials that are given by $U(x)$.

To derive the equations of motion of the amplitude and the phase, we introduce the Lagrangian of the CDW as

$$\mathcal{L} = \mathcal{T} - \mathcal{F}. \tag{5}$$

Here, $\mathcal{T}$ is the kinetic energy density which is defined as

$$\mathcal{T} = m_A\frac{\dot{A}^2}{2} + m_\varphi\frac{\dot{\varphi}^2}{2} \;. \tag{6}$$

where m_A and m_φ are the amplitude and the phase virtual masses. The equations of motion of the position-dependent amplitude and phase can be derived using this Lagrangian and the field-theoretical Euler-Lagrange formalism:

$$\ddot{A} = \frac{1}{m_A}\left(-2aA - 4bA^3 - 2c_\varphi A(\partial_x\varphi)^2 - U\cos(q_0x + \varphi) + 2c_A(\partial_x A)^2 - \gamma_A\dot{A}\right) \tag{7}$$

and

$$\ddot{\varphi} = \frac{1}{m_\varphi}\left(UA\sin(q_0x + \varphi) + 2c_\varphi(2A\cdot\partial_x A\cdot\partial_x\varphi + A^2\partial_x^2\varphi) - \gamma_\varphi\dot{\varphi} + \kappa E\right). \tag{8}$$

We added empirical damping terms that act on the phase velocity with strength γ_φ and on the amplitude velocity with strength γ_A to account for dissipation in the charge-density wave dynamics. The term κE accounts for the acceleration of the CDW phase that is caused by the excitation with the THz pulse and will be discussed in the following.

The measurements discussed in Fig. 3 and the main text show that excitation of the CDW by the THz pump pulse is caused by the THz electric field and not by THz-induced tunneling. This excitation is modeled here as a screening current that flows in the surface of the sample (see above section 2 - Screening current calculation). We consider ultrafast Joule heating and acceleration of mobile charges as the two dominant processes with which the screening current can impact the CDW dynamics. These excite both, the amplitude and phase of the CDW.

Ultrafast Joule heating causes a time-dependent change of the free energy functional that can be accounted for by a time-dependent reduction and recovery of $a = a(t)$[24,25]. This is analogous to the time- and temperature dependent $a(T, t)$ that is typically used to describe the phase transition

between CDW and normal metal state[18] as well as ultrafast laser excitation of amplitude oscillations[26] within Ginzburg Landau theory.

The in-plane screening current is a consequence of the metallic $NbSe_2$ surface screening the THz electric field in the tunnel junction. As the CDW in $NbSe_2$ is incommensurate with the host lattice, it can carry current as well. Therefore, the time-dependent acceleration of charges, that causes the screening current, must be considered in the equation of motion of the CDW. It enters directly as an acceleration that is proportional to the in-plane electric field, $\kappa E(t)$. For simplicity we assume here that this field is proportional to the far-field strength of the THz pulses and the field strength to be spatially constant within the space for which the equations of motion are solved.

3.1 Model parameters.

The equations of motion shown in eqs. (7, 8) are, in principle, applicable to any incommensurate CDW. Hence, the parameters must be adjusted to the characteristics of $NbSe_2$, as discussed in the following.

The pinning potential is modeled to represent the random distribution of atomic defects observed in STM images. We model the pinning potential of each defect as a Gaussian-shaped potential of the form $U_n(x) = -U_0 \exp(-(x - x_{0,n})^2/w^2)$, where $x_{0,n}$ is the position of the defect. We set the pinning potential's strength to U_0 = 125 J/C and its width to w = 0.1 nm. This reproduces a slightly increased local charge density as observed on many defects in our experiment. We find that the exact shape and width of the atomic pinning potentials has little impact on the CDW dynamics as long as their width is below the CDW wavelength. For simplicity, we assume that all defects have the same pinning potential, because we are primarily interested to model the impact of spatial distributions of defects on the observed dynamics rather than a quantitative modeling of individual pinning sites. Then, the pinning potential landscape is $U(x) = \sum_n U_n(x)$ with a random distribution of defects with an average density of 0.4 defects per CDW wavelength.

The time-dependent electric field that accelerates the charge density wave is assumed to follow the electric field waveform of the THz pulse as determined by electro-optic sampling in the far field, Extended Data Fig. 7. This assumption is justified because the THz pulses used in our experiment have a bandwidth of less than 1.5 THz. Attenuation and antenna effects that may change the waveform upon coupling to the tunnel junction become significant only for higher bandwidth pulses[27]. The strength of the acceleration is determined by the prefactor κ, that is, a priori, unknown. However, we found that it has very little impact on the dynamics observed after the THz pulse has passed beyond 2 ps and we set it to $\kappa = 0.14$.

To fit the numerical model to the dynamics observed on 2H-$NbSe_2$, we set the parameters of the free energy density $\tilde{a}$, b, c_A, as well as the strength of the amplitude damping, γ_A, such that our model reproduces the characteristic frequency of 1.2 THz[26] and relaxation time of the amplitude mode in $NbSe_2$ as observed by optical spectroscopy[28]. This sets an upper bound for phase excitations. The remaining free parameters were adjusted to reproduce phase excitations in the same frequency interval between 0.15 THz to 0.8 THz as was observed experimentally. Table S1 gives a list of all parameters of the model that were chosen for the calculations shown in Fig. 4.

3.2 Group velocity of phase excitations.

From equation (8), the group velocity of phase excitations in the CDW can be determined analytically in the approximation of small phase damping:

$$v_{\varphi} = |A|\sqrt{\frac{2c_{\varphi}}{m_{\varphi}}} \tag{9}$$

This velocity is determined by the input parameters of the free energy density, primarily the phase stiffness c_{φ}, and the magnitude of the order parameter, A. For the model parameters that reproduce the phase excitations between 0.15 THz to 0.8 THz (Table S1), this yields a group velocity of phase excitations of 3.2 nm/ps. This speed is comparable to the speed of sound in $NbSe_2$[29] and also consistent with predicted velocity of phasons[21].

Table S1. Simulation parameters for Fig. 4d,e.

parameter	value	unit
$\tilde{a}$	100	$\mathrm{J \cdot m/(}K\mathrm{ \cdot C^2)}$
b	0.2	$\mathrm{J \cdot m^3/C^4}$
m_A	1.0×10^{-22}	$\mathrm{kg \cdot m^3/C^2}$
c_φ	1.1×10^{-19}	$\mathrm{J \cdot m^3/C^2}$
U_0	125	J/C
w	0.1	nm
$\tilde{q}_0$	5.9	rad/nm
c_A	2.0×10^{-18}	$\mathrm{J \cdot m^3/C^2}$
γ_A	2.4×10^{-10}	$\mathrm{kg \cdot m^3/(C^2 \cdot s)}$
κ	0.14	$\mathrm{kg \cdot m^2/(V \cdot s^2)}$
γ_φ	4×10^{-11}	$\mathrm{kg \cdot m/s}$
m_φ	1.1×10^{-22}	$\mathrm{kg \cdot m}$

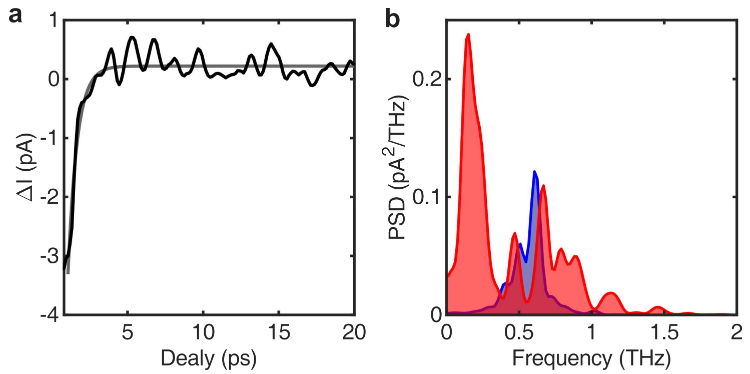

Supplementary Fig. S1 | Exponential fit of the fast decay and PSD of the time traces measured in the CDW phase. a, Difference (black curve) between the measured time trace of the THz-induced tunnel current and the calculated pulse-pulse correlation signal shown in Extended Data Fig. 2b, and the exponential fit (gray curve) which yields a decay time of $\tau = 603$ fs $\pm$ 80 fs. **b**, Comparison of the power spectral density, PSD, of the time trace measured in the CDW phase (red) and the calculated pulse-pulse correlation signal (blue) shown in Extended Data Fig. 2b. FFT time span from 3.6 ps to 23.3 ps.

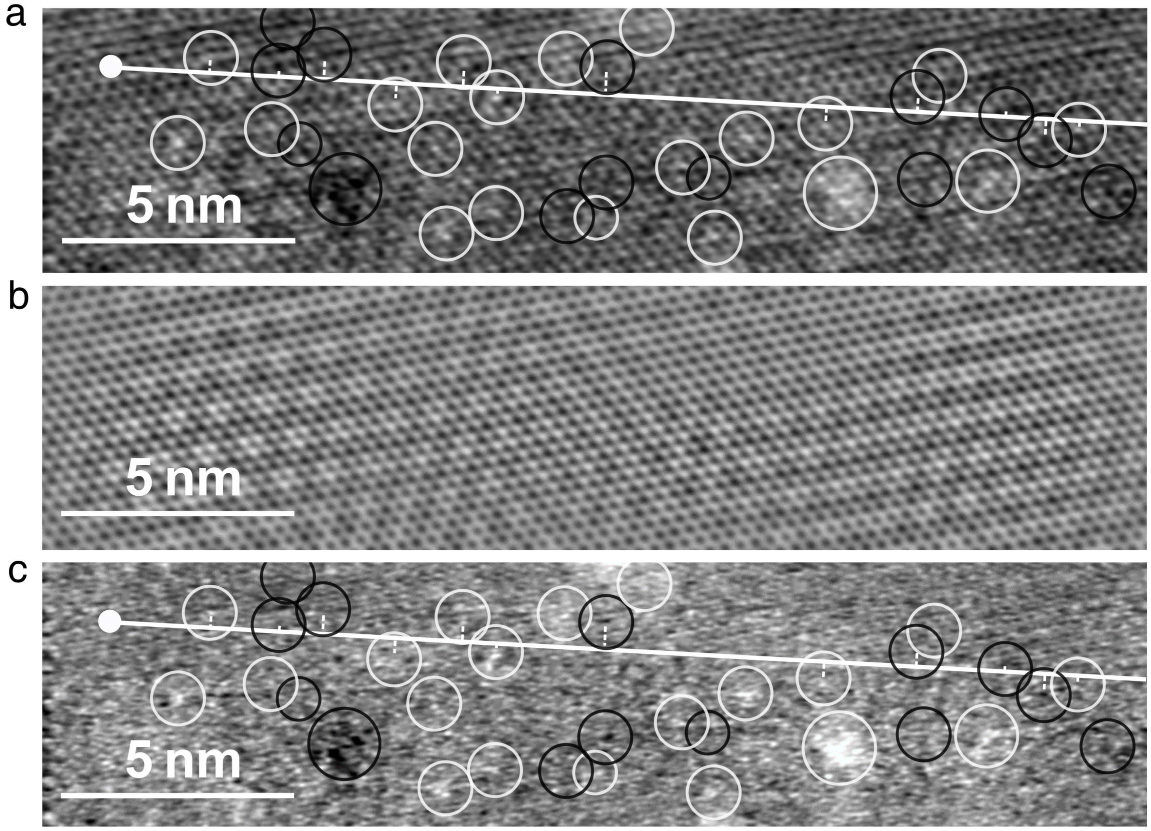

Supplementary Fig. S2 | Enlarged plot of the constant-current topography shown in Fig. 2a. **a**, Constant current topography in the CDW phase of 2H-$NbSe_2$, same as in Fig. 2a. The junction set point 12 pA at 28 mV was chosen to show defects as protrusions or depressions in the topographic signal while keeping the bias close to the set point used in Fig. 2b. Visible defects are highlighted by white circles (protrusions) and black circles (depressions). The spatially-resolved PSD shown in Fig. 2b is recorded along the white line. Positions of defects are projected onto the scanned line and indicated by white dashed lines. **b**, Fourier-filtered STM image of (a) showing only the atomic and CDW superlattice signal of Fig. 2b. The filtered image was obtained by 2D fast Fourier transform (FFT) of the STM topography (a). A filter function is applied in Fourier space that isolates the wave vector peaks of the atomic lattice and CDW wave vectors. Inverse Fourier transform of the filtered FFT yields the periodic lattice and CDW signal that is contained in the topography and highlights the spatial variations of the CDW. **c**, Non-periodic signal contained in the topography, which is primarily attributed to the presence of defects. It is obtained by subtracting the Fourier-filtered STM image (b) from the raw topography (a). This image aids in identifying defect locations in the unfiltered topography (a).

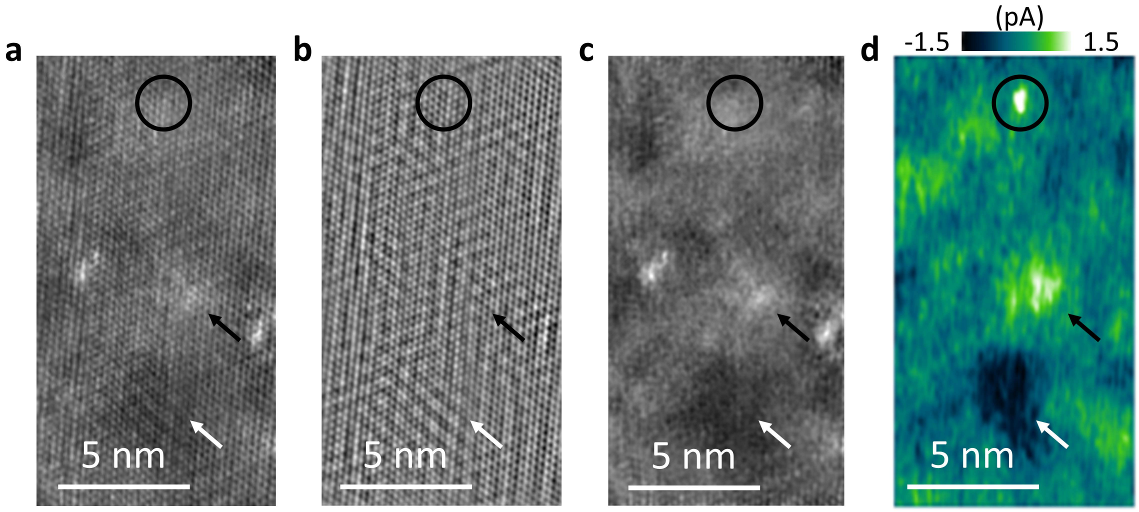

Supplementary Fig. S3 | Fourier filtered STM images for the topography shown in Fig. 2c. **a**, Constant-current topography, same as Fig. 2c in the main text. **b**, Fourier filtered STM image of (a) obtained by inverse Fourier transform of the atomic lattice and CDW supperlattice wave vector peaks in Fourier space. See Fig. S2b for details on the FFT filtering method. **c**, The difference between (a) and the filtered image (b). **d**, Map of THz-induced tunnel current recorded simultaneously with (a) same as Fig. 2d in the main text. Junction set point 1 nA at 1 mV.

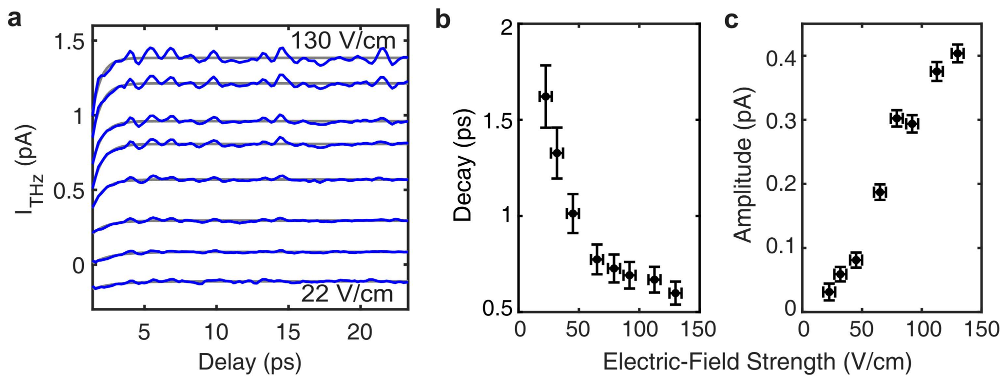

Supplementary Fig. S4 | THz electric field dependent CDW excitation dynamics. **a**, Variation of the difference signal between the measurements (Fig. 3a) and the calculated pule-pulse correlation signal with increasing electric field strength of the excitation pulse (blue lines) and exponential fits (gray lines). Electric field strength increases from bottom to top. The curves are vertically shifted for clarity. **b**,**c**, Decay constant (b), and amplitude (c) of the exponential decay for the exponential fits shown in (a) plotted as a function of excitation pulse electric field strength. The horizontal error bars in (b) and (c) represent uncertainties of the measured THz electric field. The vertical error bars in (b) and (c) correspond to 0.65 confidence level derived from the fit.

Supplementary references

1. Plankl, M. *et al.* Subcycle contact-free nanoscopy of ultrafast interlayer transport in atomically thin heterostructures. *Nat. Photonics* **15**, 594–600 (2021).
2. Stinson, H. T. *et al.* Imaging the nanoscale phase separation in vanadium dioxide thin films at terahertz frequencies. *Nat. Commun.* **9**, 1–9 (2018).
3. Naftaly, M. & Gregory, A. Terahertz and microwave optical properties of single-crystal quartz and vitreous silica and the behavior of the boson peak. *Appl. Sci.* **11**, 6733 (2021).
4. Podzorov, A. & Gallot, G. Low-loss polymers for terahertz applications. *Appl. Opt.* **47**, 3254–3257 (2008).
5. Liu, J., Dai, J., Chin, S. L. & Zhang, X. C. Broadband terahertz wave remote sensing using coherent manipulation of fluorescence from asymmetrically ionized gases. *Nat. Photonics* **4**, 627 (2010).
6. Abdo, M. *et al.* Variable repetition rate THz source for ultrafast scanning tunneling microscopy. *ACS Photonics* **8**, 702–708 (2021).
7. Cocker, T. L. *et al.* An ultrafast terahertz scanning tunnelling microscope. *Nat. Photonics* **7**, 620–625 (2013).
8. Ammerman, S. E., Wei, Y., Everett, N., Jelic, V. & Cocker, T. L. Algorithm for subcycle terahertz scanning tunneling spectroscopy. *Phys. Rev. B* **105**, 115427 (2022).
9. Sheng, S. *et al.* Launching coherent acoustic phonon wavepackets with local femtosecond Coulomb forces. *Phys. Rev. Lett.* **129**, 043001 (2022).
10. Dordevic, S. V., Basov, D. N., Dynes, R. C. & Bucher, E. Anisotropic electrodynamics of layered metal 2H-$NbSe_2$. *Phys. Rev. B* **64**, 161103 (2001).
11. Sadeghi, A., Baratoff, A. & Goedecker, S. Electrostatic interactions with dielectric samples in scanning probe microscopies. *Phys. Rev. B* **88**, 035436 (2013).
12. Reimann, J. *et al.* Subcycle observation of lightwave-driven Dirac currents in a topological surface band. *Nature* **562**, 396–400 (2018).
13. Murphy, B. M. *et al.* Phonon modes at the 2H-$NbSe_2$ surface observed by grazing incidence inelastic X-ray scattering. *Phys. Rev. Lett.* **95**, 256104 (2005).
14. Vaskivskyi, I. *et al.* Fast electronic resistance switching involving hidden charge density wave states. *Nat. Commun.* **7**, 11442 (2016).
15. Hohenberg, P. C. & Krekhov, A. P. An introduction to the Ginzburg-Landau theory of phase transitions and nonequilibrium patterns. *Phys. Rep.* **572**, 1–42 (2015).
16. Maklar, J. *et al.* Nonequilibrium charge-density-wave order beyond the thermal limit. *Nat. Commun.* **12**, 2499 (2021).
17. Zhou, F. *et al.* Nonequilibrium dynamics of spontaneous symmetry breaking into a hidden state of charge-density wave. *Nat. Commun.* **12**, 566 (2021).
18. Grigorishin, K. V. Extended time-dependent Ginzburg–Landau theory. *J. Low Temp. Phys.* **203**, 262–308 (2021).
19. Thomson, M. D. *et al.* Phase-channel dynamics reveal the role of impurities and screening in a quasi-one-dimensional charge-density wave system. *Sci. Rep.* **7**, 2039 (2017).
20. Danz, T., Domröse, T. & Ropers, C. Ultrafast nanoimaging of the order parameter in a structural phase transition. *Science* **371**, 371–374 (2021).
21. Grüner, G. The dynamics of charge-density waves. *Rev. Mod. Phys.* **60**, 1129–1181 (1988).
22. Van Bakel, G. P. E. M. & De Hosson, J. T. M. Various regimes of charge-density waves in layered compounds. *Phys. Rev. B* **46**, 2001–2007 (1992).

23. Oh, E., Gye, G. & Yeom, H. W. Defect-selective charge-density-wave condensation in 2H-$NbSe_2$. *Phys. Rev. Lett.* **125**, 036804 (2020).
24. Gor'kov, L. P. & Grüner, G. *Charge density waves in solids*. (Elsevier, 2012).
25. McMillan, W. L. Theory of discommensurations and the commensurate-incommensurate charge-density-wave phase transition. *Phys. Rev. B* **14**, 1496–1502 (1976).
26. Méasson, M. A. *et al.* Amplitude Higgs mode in the 2H-$NbSe_2$ superconductor. *Phys. Rev. B* **89**, 60503 (2014).
27. Müller, M., Sabanés, N. M., Kampfrath, T. & Wolf, M. Phase-resolved detection of ultrabroadband THz pulses inside a scanning tunneling microscope junction. *ACS Photonics* **7**, 2046 (2020).
28. Anikin, A. *et al.* Ultrafast dynamics in the high-symmetry and in the charge density wave phase of 2H-$NbSe_2$. *Phys. Rev. B* **102**, 205139 (2020).
29. Jericho, M. H., Simpson, A. M. & Frindt, R. F. Velocity of ultrasonic waves in 2H-$NbSe_2$, 2H-TaS_2, and 1T-TaS_2. *Phys. Rev. B* **22**, 4907–4914 (1980).
